# Pharmacophore modeling, 2D-QSAR, drug likeness and toxicity prediction of 2-aziridinyl- and 2,3-bis(aziridinyl)-1,4-naphthoquinonyl sulfonate and acylate derivatives as multifunctional agents for the treatment of malaria parasite, *Plasmodium falciparum*

**DOI:** 10.1016/j.namjnl.2025.100019

**Published:** 2025-04-17

**Authors:** Emmanuel Israel Edache, Fabian Audu Ugbe, Hadiza Adamu Dawi, Adebiyi Adedayo, Ahmed Umar, Aqel Albutti

**Affiliations:** a Department of Pure and Applied Chemistry, University of Maiduguri, Maiduguri, Borno State, Nigeria; b Department of Chemistry, University of Abuja, Gwagwalada, FCT Abuja, Nigeria; c Department of Pharmaceutical and Medicinal Chemistry/ Drug Development, Sheda Science and Technology Complex, FCT Abuja, Nigeria; d Department of Basic health Sciences, College of Applied Medical Sciences, Qassim University

**Keywords:** Pharmacophoric, Docking, ADMET, MD simulations, Malaria

## Abstract

Pharmacophore modeling, QSAR, molecular docking, in silico ADME, and toxicity prediction have been conducted on 2-aziridinyl- and 2,3-bis(aziridinyl)-1,4-naphthoquinonyl sulfonate and acylate-based malaria parasite inhibitors. In this study, we aim to clarify the binding mode and drug-likeness of these derivatives. Ultimately, 63 compounds were utilized to develop a model. The model was statistically significant, exhibiting a high correlation coefficient (R² = 0.8066), a cross-validation coefficient (Q²_loo_ = 0.7559), and an F value of 43.3149. The results from external validation also demonstrate strong predictive power (R²_ext_ = 0.7325). In addition to passing Tropsha's test for predictive ability, the resulting model also successfully passed the Y-randomization test. The model’s applicability domain (AD) was clearly defined, indicating that in the case of a new prediction, it is evident whether that prediction can be safely made. To assess the impact of the docking protocol, a co-crystallized ligand from the protein's ligand-binding domain was selected and re-docked. The results after redocking and the co-crystallized ligand were aligned, with a mean root-mean-square deviation along their lines of 1.703 Å. Using the DTM protein co-crystal ligand (PDB ID: 1MVT) as a template, the pharmacophore hypotheses were produced. A screening of PubChem databases resulted in 40 hits that passed the screening requirements for docking against the DTM receptor through the Pharmit server. The top two docked compounds having the highest binding affinities from -11.5 to -11.1 kcal/mol were selected. A further ADMET analysis showed that the selected compounds have values within the acceptable range. Molecular dynamics simulation analysis of inhibitor potentials against the malaria enzyme, compound 18 has strong inhibitory potential in treating malaria diseases. Findings from this study inform on possible ways of designing new inhibitors for malaria parasites.

## 1 Introduction

Humans and other animals can contract malaria, an infectious disease spread by mosquitoes. It is caused by five species of Plasmodium, which are transmitted through bites from female Anopheles mosquitoes. Symptoms of malaria include fever, headaches, and vomiting (Zambare et al., 2019). With over 500,000 deaths annually worldwide, malaria continues to be one of the leading diseases impacting the most vulnerable groups, despite its high cost (Rogerson et al., 2020). According to a WHO report, 219 million cases of malaria were reported in 87 countries in 2017, with an estimated 435,000 people dying from the disease. Despite this, there is currently no practical vaccination against malaria parasites (Nnamonu et al., 2020; Talapko et al., 2019). The lack of a highly effective vaccine and treatment resistance makes malaria a very difficult disease that poses a significant public health burden. Chloroquine, Chloroguanide (Proguanil), Sulfadoxine/pyrimethamine (SP), Quinine, Mefloquine, Halofantrine, and Atovaquone were the most widely used antimalarial medications used to treat malarial parasites (Grabias and Kumar 2016). The development of novel antimalarial medications with distinct mechanisms of action and the identification of new or innovative drug targets may provide a solution to drug resistance in malaria, which in turn may reduce malarial mortality (Tse et al., 2019). Numerous possible therapeutic targets have been suggested in research because these disorders are extensively explored; these targets can also be developed as drug targets in trials. The literature also reported on the Ras-related protein Rab-6, which may be used as a therapeutic target or vaccine candidate for many pathogen diseases (Vandana and Jaiswal, 2020). Ras-related protein is expressed in nearly every stage of *Plasmodium falciparum* in humans, which supports its significance in the *Plasmodium* life cycle and its potential as a therapeutic target (Guleria and Jaiswal, 2020).

To investigate how 2-aziridinyl- and 2,3-bis(aziridinyl)-1,4-naphthoquinonyl sulfonate and acylate derivatives block the enzyme of the malaria parasite *Plasmodium falciparum*, we conducted in silico modeling in this work. The research encompasses molecular docking, pharmacophore modeling, molecular dynamics (MD) simulations, molecular mechanics/generalized Born surface area (MM/PB-GBSA) computations, and quantitative structure-activity relationship (QSAR). To study the protein-ligand complex, calculations were made using the *Plasmodium falciparum* enzyme, which is a parasite that causes malaria. The ligand binding free energies of 2-aziridinyl- and 2,3-bis(aziridinyl)-1,4-naphthoquinonyl sulfonate and acylate derivatives, as determined using the MM/PB(GB)SA method, and the malaria parasite *Plasmodium falciparum* enzyme conformation changes suggested by the MD simulations would validate the dependencies of site activity on the electrostatic and hydrophobic properties of the ligands. Finding the physicochemical characteristics of some 2-aziridinyl- and 2,3-bis(aziridinyl)-1,4-naphthoquinonyl sulfonate and acylate derivatives that are used to treat *Plasmodium falciparum*, the malaria parasite, was the goal of this study.

## 2 Materials and methods

### 2.1 Ligand preparation

The PubChem database (https://pubchem.ncbi.nlm.nih.gov/bioassay/152094) provided the SMILES (Simplified Molecular Input Line Entry Specification) format of 2-aziridinyl- and 2,3-bis(aziridinyl)-1,4-naphthoquinonyl sulfonate and acylate derivatives of antimalarial activity (Table 1). Marvin-Sketch software (https://www.chemaxon.com) was used to convert the SMILES formats to 2D SD file structures, and the MzDock software package was used to optimize energy minimization (Kabier et al., 2024). The MMFF94 field was used for energy minimization and geometry optimization (Halgren, 1996). To find structural analogs of antimalarial action, the QSAR analysis and high throughput screening used pIC_50_ (-logIC_50_) values to reduce the skewness of the data set, which were derived from the inhibitory activity represented as IC_50_ values. The pIC_50_ values, which span a range of three log units from 5.1192 to 7.6198, show that the range required to produce trustworthy QSAR models has been attained.

**Table 1:** Molecules in SMILES format with their pIC_50_ values.

S/N	CID	Canonical SMILES	pIC50
1	4055	O=C1C=C(C)C(=O)c2c1cccc2	5.4685
2	14,460,433	O=C1C(=C(N2CC2)C(=O)c2c1c(ccc2)OS(=O)(=O)c1ccccc1)N1CC1	5.2366
3	44,282,886	Brc1ccc(cc1)S(=O)(=O)Oc1cccc2c1C(=O)C(=C(C2=O)N1CC1)N1CC1	5.4318
4	14,460,438	Clc1ccc(cc1)S(=O)(=O)Oc1cccc2c1C(=O)C(=C(C2=O)N1CC1)N1CC1	5.4949
5	14,460,437	O=C1C(=C(N2CC2)C(=O)c2c1c(ccc2)OS(=O)(=O)c1c(cc(cc1C(C)C)C(C)C)C(C)C)N1CC1	5.5229
6	14,460,436	Cc1cc(C)c(c(c1)C)S(=O)(=O)Oc1cccc2c1C(=O)C(=C(C2=O)N1CC1)N1CC1	5.4089
7	129,237	O=C1C(=CC(=O)c2c1cccc2O)N1CC1	7.0555
8	44,282,905	CCc1ccc(cc1)S(=O)(=O)Oc1cccc2c1C(=O)C(=C(C2=O)N1CC1)N1CC1	5.3979
9	14,460,439	O=C1C(=C(N2CC2)C(=O)c2c1c(ccc2)OS(=O)(=O)c1ccc(cc1)[N+](=O)[O-])N1CC1	5.1192
10	44,282,909	Ic1ccc(cc1)S(=O)(=O)Oc1cccc2c1C(=O)C(=C(C2=O)N1CC1)N1CC1	5.3872
11	14,460,435	O=C1C(=C(N2CC2)C(=O)c2c1c(ccc2)OS(=O)(=O)c1ccc(cc1)C(C)(C)C)N1CC1	5.3979
12	14,460,431	Oc1cccc2c1C(=O)C(=C(C2=O)N1CC1)N1CC1	5.7959
13	14,590,284	Cc1ccc(cc1)S(=O)(=O)Oc1cccc2c1C(=O)C=C(C2=O)N1CC1	6.699
14	14,460,432	Cc1ccc(cc1)S(=O)(=O)Oc1cccc2c1C(=O)C(=C(C2=O)N1CC1)N1CC1	5.3372
15	14,460,446	ClCCCS(=O)(=O)Oc1cccc2c1C(=O)C(=C(C2=O)N1CC1)N1CC1	5.5229
16	14,460,444	O=C1C(=C(N2CC2)C(=O)c2c1c(ccc2)OS(=O)(=O)C)N1CC1	5.4949
17	14,460,443	O=C1C(=C(N2CC2)C(=O)c2c1c(ccc2)OS(=O)(=O)c1cccc2c1cccc2N(C)C)N1CC1	5.5229
18	14,460,441	O=C1C(=C(N2CC2)C(=O)c2c1c(ccc2)OS(=O)(=O)c1cccc2c1cccc2)N1CC1	5.4685
19	14,590,296	O=C1C(=C(N2CC2)C(=O)c2c1c(ccc2)OS(=O)(=O)Cc1ccccc1)N1CC1	5.6198
20	14,460,447	O=C1C(=C(N2CC2)C(=O)c2c1c(ccc2)OS(=O)(=O)/C=C/c1ccccc1)N1CC1	5.2366
21	14,460,448	O=C1CC2C(C1(CC2)CS(=O)(=O)Oc1cccc2c1C(=O)C(=C(C2=O)N1CC1)N1CC1)(C)C	5.2218
22	14,867,494	O=C1C(=C(N2CC2)C(=O)c2c1cccc2O)C	5.8239
23	14,867,505	O=C1C=C(N2CC2)C(=O)c2c1c(ccc2)OS(=O)(=O)c1cccc2c1nccc2	6.3665
24	14,867,504	O=C1C=C(N2CC2)C(=O)c2c1c(ccc2)OS(=O)(=O)c1cccc2c1cccc2N(C)C	6.5376
25	44,289,070	O=C1C(=C(N2CC2)C(=O)c2c1c(ccc2)OS(=O)(=O)c1ccc(c(c1)[N+](=O)[O-])[N+](=O)[O-])N1CC1	5.6198
26	14,867,506	O=C1C=C(N2CC2)C(=O)c2c1c(ccc2)OS(=O)(=O)c1cccs1	6.3279
27	14,867,496	ClCCNC1=CC(=O)c2c(C1=O)cccc2O	5.3665
28	14,867,502	O=C1C=C(N2CC2)C(=O)c2c1c(ccc2)OS(=O)(=O)c1c(cc(cc1C(C)C)C(C)C)C(C)C	6.5528
29	10,205	CC1=CC(=O)c2c(C1=O)cccc2O	5.6198
30	14,867,518	COc1ccc(cc1)C(=O)Oc1cccc2c1C(=O)C(=C(C2=O)N1CC1)N1CC1	5.4318
31	14,867,495	CNC1=CC(=O)c2c(C1=O)cccc2O	5.4318
32	14,867,520	O=C1C(=C(N2CC2)C(=O)c2c1c(ccc2)OC(=O)c1ccco1)N1CC1	5.3372
33	14,867,517	Clc1ccc(cc1)C(=O)Oc1cccc2c1C(=O)C(=C(C2=O)N1CC1)N1CC1	5.7696
34	14,867,523	O=C(C12CC3CC(C2)CC(C1)C3)Oc1cccc2c1C(=O)C(=C(C2=O)N1CC1)N1CC1	5.2676
35	14,867,508	Cc1ccc(cc1)C(=O)Oc1cccc2c1C(=O)C=C(C2=O)N1CC1	6.2076
36	14,867,500	Ic1ccc(cc1)S(=O)(=O)Oc1cccc2c1C(=O)C=C(C2=O)N1CC1	6.4815
37	14,867,507	O=C1C=C(N2CC2)C(=O)c2c1c(ccc2)OC(=O)c1ccccc1	6.3468
38	14,867,503	O=C1C=C(N2CC2)C(=O)c2c1c(ccc2)OS(=O)(=O)c1cccc2c1cccc2	6.8861
39	14,867,498	COc1ccc(cc1)S(=O)(=O)Oc1cccc2c1C(=O)C=C(C2=O)N1CC1	6.7959
40	131,581	CCc1ccc(cc1)S(=O)(=O)Oc1cccc2c1C(=O)C=C(C2=O)N1CC1	7.0177
41	14,867,527	ClCCNC1=CC(=O)c2c(C1=O)cccc2OS(=O)(=O)c1cccc2c1cccc2N(C)C	5.5376
42	14,867,525	ClCCNC1=CC(=O)c2c(C1=O)cccc2OS(=O)(=O)N(C)C	5.6576
43	3806	O=C1C=CC(=O)c2c1c(O)ccc2	5.4318
44	14,034,881	CN(C1=CC(=O)c2c(C1=O)cccc2O)C	5.7959
45	131,582	O=C1C=C(N2CC2)C(=O)c2c1c(ccc2)OS(=O)(=O)c1ccc(cc1)C(C)(C)C	7.6198
46	14,867,499	Fc1ccc(cc1)S(=O)(=O)Oc1cccc2c1C(=O)C=C(C2=O)N1CC1	6.8539
47	14,867,501	O=C1C=C(N2CC2)C(=O)c2c1c(ccc2)OS(=O)(=O)/C=C/c1ccccc1	6.7696
48	14,867,516	Fc1ccc(cc1)C(=O)Oc1cccc2c1C(=O)C(=C(C2=O)N1CC1)N1CC1	5.3565
49	14,867,515	Cc1ccc(cc1)C(=O)Oc1cccc2c1C(=O)C(=C(C2=O)N1CC1)N1CC1	5.3565
50	14,867,497	O=C1C=C(N2CC2)C(=O)c2c1c(ccc2)OS(=O)(=O)c1ccccc1	6.6383
51	10,380,617	O=C1C=C(N2CC2)C(=O)c2c1c(ccc2)OC(=O)c1ccco1	6.3098
52	14,867,512	COc1cc(cc(c1OC)OC)C(=O)Oc1cccc2c1C(=O)C=C(C2=O)N1CC1	6.7959
53	14,867,511	COc1ccc(cc1)C(=O)Oc1cccc2c1C(=O)C=C(C2=O)N1CC1	6.2676
54	14,867,510	Clc1ccc(cc1)C(=O)Oc1cccc2c1C(=O)C=C(C2=O)N1CC1	6.2757
55	14,867,514	O=C1C(=C(N2CC2)C(=O)c2c1c(ccc2)OC(=O)c1ccccc1)N1CC1	5.585
56	14,867,513	O=C1C=C(N2CC2)C(=O)c2c1c(ccc2)OC(=O)c1cccs1	6.3279
57	14,867,509	Fc1ccc(cc1)C(=O)Oc1cccc2c1C(=O)C=C(C2=O)N1CC1	6.3098
58	14,867,528	O=C1C(=C(N2CC2)C(=O)c2c1cccc2OS(=O)(=O)c1ccc(cc1)C(C)(C)C)C	5.4437
59	14,867,522	Clc1sc(c(c1)C(=O)Oc1cccc2c1C(=O)C(=C(C2=O)N1CC1)N1CC1)Cl	5.4089
60	14,867,521	O=C1C(=C(N2CC2)C(=O)c2c1c(ccc2)OC(=O)c1cccs1)N1CC1	5.585
61	14,867,524	ClCCNC1=CC(=O)c2c(C1=O)cccc2OS(=O)(=O)C	5.0706
62	14,867,526	ClCCNC1=CC(=O)c2c(C1=O)cccc2OS(=O)(=O)Cc1ccccc1	5.1871
63	14,867,519	COc1cc(cc(c1OC)OC)C(=O)Oc1cccc2c1C(=O)C(=C(C2=O)N1CC1)N1CC1	5.4685

For a limited period, the chemical descriptors of the data set were calculated using the Pharmaceutical Data Exploration Laboratory (PaDEL-Descriptor) Software v2.21 (Yap, 2011). Since molecular descriptors are used to describe the different characteristics of compounds and support structural information analysis, they are often extremely important for QSAR research.

### 2.2 QSAR calculation

Gramatica et al.'s QSARINS v2.2.4 software was used to do the QSAR analysis (Gramatica et al., 2013, 2014). A genetic algorithm (GA) assisted in the selection of the descriptors, while multiple regression was used as the regression technique. The population size of 100 and the mutation rate of 25 % were the set of parameters used to manage GA. Two groups were created from the analyzed derivatives: the training group (*n* = 46, ≈ 70 %) and the testing group (*n* = 17, ≈ 30 %). Before QSAR analysis, the data division was random. The coefficient of determination (R^2^), predictive squared correlation coefficient (Q^2^_loo_), and root-mean-squared error of cross-validation (RMSE_CV_) derived from the leave-one-out cross-validation approach were used to evaluate the model's fitting, robustness, and predictive capabilities. The Q^2^ must be higher than 0.5 for the 2D-QSAR model to be considered acceptable (Ugbe et al., 2022a, Ugbe et al., 2022b). To prevent chance association between the antiproliferative activity in the chosen QSAR model and the retained descriptors, the "Y-randomization test" (Edache et al., 2015) was also investigated. Additionally, the external validation-derived root-mean-square error in prediction (RMSEP) was computed.

(1) $$Q^{2}=1-\left[\frac{\sum {\left(Y_{train}-Y_{pred\left(LOO\right)}\right)}^{2}}{\sum {\left(Y_{train}-{\bar{Y}}_{LOO}\right)}^{2}}\right]$$

(2) $$R_{pred}^{2}=1-\left[\frac{\sum {\left(Y_{test}-Y_{pred\left(test\right)}\right)}^{2}}{\sum {\left(Y_{test}-Y_{train}\right)}^{2}}\right]$$

Here, $Y_{train}$ and $Y_{pred}$ are respectively the experimental and predicted pIC_50_ values of each molecule (training set/test set), representing the average pIC_50_ values of the molecules in the training and test set, respectively.

### 2.3 Applicability domain (AD)

In silico predictions of the activities of new compounds should be validated under OECD QSAR validation guidelines (Voigt and Jaeger, 2023). The chemical space in which new compounds' biological activity might be reliably predicted is known as the AD of the established QSAR model (Abechi and Edache, 2016). Only when the anticipated chemical falls inside the scope of application is the prediction deemed trustworthy. William plot analysis and the Insubria plot were used to look for any outliers in the data set. The standardized residuals are shown here in proportion to leverage. In this instance, the compounds significantly affect the anticipated model if the high leverage (h) is higher than the warning leverage (h*) (Edache et al., 2016a; Ugbe et al., 2022a, Ugbe et al., 2022b).

### 2.4 Protein preparation

**Fig. 1:**
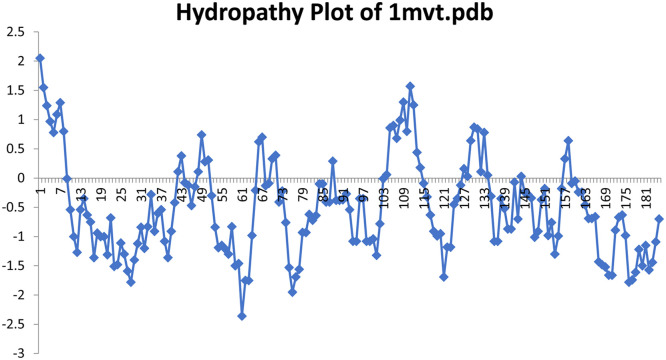
The transmembrane region of 1MVT.pdb was predicted using the hydropathy plot.

Human dihydrofolate reductase was chosen as the target protein (PDB code:1MVT; resolution 1.80 Å; R-value free 0.194, R-value work 0.2160). The protein target was the crystal structure of analysis of two polymorphic forms of a pyrido[2,3-d]pyrimidine N9-C10 reverse-bridge antifolate binary complex. The protein was downloaded from https://www.rcsb.org/structure/1MVT. Fig. 1 displays the hydropathy plot of 1MVT and FASTA format of the input PDB file. It is more hydrophobic if the total hydrophobicity value is greater than zero. The region's hydrophilic inclination is shown by negative values. The complex was shown in the macromolecule view using Dockey v1.0.3 (Du et al., 2023), which was used to automatically activate the PDB interpretation method.

### 2.5 Molecular docking

All ligands in the peptidase domain of two polymorphic variants of a pyrido[2,3-d] pyrimidine N9-C10 reverse-bridge antifolate binary complex with the human dihydrofolate reductase receptor were subjected to molecular docking. For molecular docking, Dockey v1.0.3 (Du et al., 2023) incorporated AutoDock-Vina v1.2.5 (Trott and Olson, 2010). *Re*-docking confirmed the docking protocol's dependability. To characterize the binding site, the native ligand (standard) was first removed from the protein binding site cavity. Current ligand selection was then used to get the ligand grid or characteristics *X* = 20, *Y* = 28, *Z* = 16, and dimension center coordinates *X* = −3.08, *Y* = 28.103, and *Z* = 2.734 for 1MVT. The interaction between its ligand and protein was examined. The best-docked model was chosen based on the lowest binding affinity, and the best-docked position was chosen by comparing the H-bond and other important interactions. The acceptability of the docking is confirmed by the RMSD of < 2 Å achieved for the superimposition of the native crystal with the best-docked posture.

### 2.6 Calculation of metrics

In the last ten years, calculated criteria have been essential in determining which medications advanced to clinical trials and were eventually brought to market (Mohs and Greig, 2017). These computed metrics serve as a guide for choosing and refining possible lead compounds in virtual screening. One of the main metrics used to assess ligand-receptor interactions is ligand efficiency (LE). Size-independent ligand efficiency (SILE) and fit quality (FQ) were proposed to alleviate the size reliance of LE (Vásquez Jiménez, 2020). Furthermore, ligand efficiency-dependent lipophilicity (LELP) and lipophilic ligand efficiency (LLE) are used to measure lipophilicity, which is a crucial component in drug development. When LogD is less than three, the ideal LLE range is five to seven or higher (Scot and Waring, 2018).

Another important factor is enzyme inhibition, which is often assessed using the inhibitory constant (Ki) and IC_50_, which represent the concentration needed to reduce enzyme activity by half. Lower Ki values indicate greater binding and more inhibitory potential, while IC_50_ indicates functional efficacy. Ki represents binding affinity. Notably, since Ki incorporates IC_50_ into its computation, pharmaceutical firms are reporting it more often. If a medicine's Ki is far greater than the highest drug concentration that patients usually experience, it is unlikely to block an enzyme (Kant et al., 2019). The permissible lower limit is defined and metrics are calculated using the formula shown in Table 2.

**Table 2:** Parameters used for ligand efficiency calculation.

S/N	Symbol	Name	Formula	Acceptable lower limit
1	LE	Ligand efficiency	-ΔG/N	≥ 0.3
2	SILE	size-Independent Ligand efficiency	-ΔG/(N^0.3^)	No value recommended by the metric originator
3	FQ	Fit Quality	LE/(0.0715+7.5328/*N* + 25.7079/N^2^−361.4722/N^3^)	≥ 0.8
4	LLE	Lipophilic ligand efficiency	-logKi - logP	∼5–7 or greater
5	LELP	Ligand Efficiency dependent Lipophilicity	logP/LE	–10 to +10
6	Ki	Estimated inhibition constant	Ki = exp(ΔG/(*R* × *T*)) The binding affinity ΔG is in kcal/mol, the universal gas constant *R* = 1.987 kcal/K/mol, at room temperature (25 °C) *T* = 273 + 25 = 298 K. Ki is having a unit of mM.	The smaller the Ki, the greater the binding affinity

NB: ΔG = free energy of binding; *N* = the number of heavy atoms (non-hydrogen atoms) of the ligand; Ki = estimated inhibition constant; LogP = the calculated octanol-water partition coefficient.

### 2.7 Generation and validation of the 3D-pharmacophore model

**Fig. 2:**
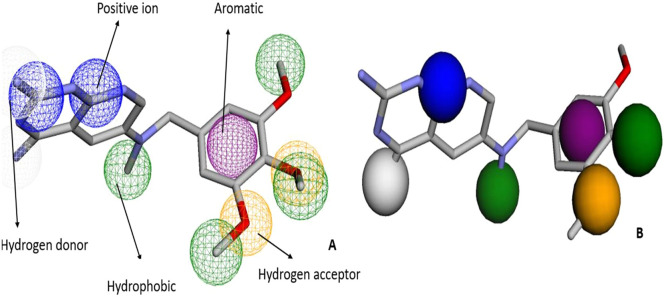
Mapping and generating 3D-pharmacophore geometry with six features of the co-crystallized ligand.

The pharmacophore model was constructed using Pharmit (http://pharmit.csb.pitt.edu). The crystalline ligand (DTM) of the human malaria parasite, *P falciparum* was utilized as a reference molecule. A 3D pharmacophore model is generated by analyzing the interaction between DTM and the active site. During the pharmacophore development process, the website made use of several properties, including hydrophobic groups, aromatic groups, positive ion groups, hydrogen bond donors, and hydrogen bond acceptors (Fig. 2A). Six properties are represented by the derived 3D pharmacophore model: two hydrophobic groups, one aromatic, one positive ion, one H-bond acceptor, and one H-bond donor in addition (Fig. 2B). To assess the tested metabolites as potential *P falciparum* inhibitors, it was used as a 3D query.

### 2.8 ADME/T analysis

The ADME/T (absorption, distribution, metabolism, excretion, and toxicity) analysis was completed for 2-aziridinyl- and 2,3-bis(aziridinyl)-1,4-naphthoquinonyl sulfonate and acylate derivatives following the molecular docking investigation. This analysis will determine whether these chemicals can be used in future in vitro and in vivo research. Using the Swiss-ADME application (www.swissadme.ch) and PKCSM (https://biosig.lab.uq.edu.au/pkcsm/prediction), the ADME/T was carried out (Daina et al., 2017).

### 2.9 Molecular dynamics (MD) simulations

A common method for evaluating an atom's behavior, structural stability, and conformational changes at the atomic level is the molecular dynamic approach (Edache et al., 2024a). To determine the dynamic characteristics of the water molecules in the malaria protein's ligand-binding pocket and to interpret its structural changes from native to various ligand-bound conformations, the initial docked structure of the human malaria parasite *Plasmodium falciparum* molecule and the native conformation of 1MVT were acquired for additional MD simulation studies. Here, the top-ranked compounds, their ADMET model, and the crystal structure of the *P falciparum* enzyme were all subjected to MD simulation using nanoscale molecular dynamics (NAMD) software (Phillips et al., 2005) at a temperature of 310 (Jo et al., 2008). The CHARMM-GUI website (http://www.charmm-gui.org/) produced the MD simulation configuration files. The CHARMM-GUI website was used for ligand parameterization. The transferable intermolecular potential water molecules (TIP3P) model was used to solve all systems (Boonstra et al., 2016). MD simulations were run in two stages: production for 200 ns timeframe under the NPT ensemble and equilibration under the NVT ensemble for 40 fs duration, where the systems' energy was minimized for 20,000 steps. Root-mean-square deviation (RMSD), the radius of gyration (RoG), root mean square fluctuation (RMSF), solvent-accessible surface area (SASA), and hydrogen bond (H-bond) were used by VMD software scripts to analyze a cube that was distributed within 20 Å on each side of the process and periodic boundary conditions were carried out during the simulation period.

### 2.10 Binding free energy calculation (MM/PB(GB)SA)

The binding free energy (ΔG_bind_) of the malaria protein-ligand complex was estimated using the molecular mechanics/Poisson-Boltzmann (General-Born) surface area (MM-PB(GB)SA) approach, a commonly used endpoint method in free energy calculations (Ugbe et al., 2024; Edache et al., 2024b). The gas phase free energy (ΔGMM), the solvation free energy (ΔGsol), and the change in the system entropy (−TΔS) all influence the binding free energy (ΔG_bind_) between protein and ligand. These can be computed using the molecular mechanics Poisson-Boltzmann Surface Area (MM/PB(GB)SA) module, as shown in Eqs. (3) to 7 (Kollman et al., 2000):

(3) ΔGbind=Gcomplex−(Gprotein+Gligand)

(4) ΔGbind=ΔEgas+ΔGsol−−TΔS

(5) ΔEgas=ΔEint+ΔEELE+ΔEVDW

(6) ΔGsol=ΔGpolar+ΔGnonpolar=ΔGGB+ΔGSA

(7) TΔS=T(ΔStrans+ΔSrot+ΔSvib

Where ΔG_bind_ refers to the total binding free energy of complex in kcal/mol which is a combination of molecular mechanics gas phase energy (ΔEgas), and solvation free energy (ΔGsol) as displayed in equation (Eq.) 4. To determine the interaction energy between the ligand and the residues of the protein active site, MM/PB(GB)SA calculations were carried out in the current work utilizing the Calculation of Free Energy (CaFE) and MOLAICAL tools (Liu and Hou, 2016; Bai et al., 2021). The reciprocal of grid spacing of 1.0 Å was used in the MM-PB(GB)SA computations, and the exterior and internal dielectric constants were set at 80.0 and 1.0, respectively. The MD trajectory was used to retrieve 100 snapshots, which were then used to calculate the MM-PB(GB)SA binding free energy (ΔGbind). In the linear interaction energy (LIE) technique, the preset coefficients α, β, and γ are defaulted to 0.18, 0.33, and 0.0, respectively. All of the docked ligands were explicitly simulated in an unbound state using the same settings as the MD simulation approach to perform the end-state LIE calculation. The linear interaction energy (LIE), as used in the LIE approach, can be expressed as:

(8) $$LIE=\alpha \left(\Delta G_{LJ,bound-unbound}\right)+\beta \left(\Delta G_{Coul,bound-unbound}\right)+\gamma$$

(9) $$\Delta G_{bind}=\alpha \left({\left[V_{LJ,\;Lig-Surr}\right]}_{bound}-{\left[V_{LJ,Lig-Surr}\right]}_{unbound}\right)+\beta \left({\left[V_{Coul,Lig-surr}\right]}_{bound}-{\left[V_{Coul,Lig-surr}\right]}_{unbound}\right)+\gamma$$

The ${\left[V_{\mathrm{LJ},\;\mathrm{Lig}-\mathrm{Surr}}\right]}_{\mathrm{bound}}$ and ${\left[V_{\mathrm{LJ},\mathrm{Lig}-\mathrm{Surr}}\right]}_{\mathrm{unbound}}$ are the average Lennard -Jone (LJ) = van der Waals (vdw) interaction energies of two complex bound or unbound statuses. The ${\left[V_{Coul,Lig-surr}\right]}_{bound}$ and ${\left[V_{Coul,Lig-surr}\right]}_{unbound}$ are the average Coulombic (Coul) = electrostatic (ele) interaction energies from two protein-ligand bound or unbounded status.

## 3 Results and discussion

### 3.1 Validation of the QSAR protocol

QSAR is one of the most widely used methods in ligand-based drug design (Edache et al., 2022). The QSAR calculation was performed using computational methods to determine the relationship between the structure and biological activities of bioactive compounds. Four descriptors, including the BCUT Descriptor, Electro-topological State Atom Type Descriptor, MDE Descriptor, and Walk Count Descriptor, were used to construct the optimal QSAR model. To link the pIC_50_ to four descriptors, the MLR analysis using a genetic algorithm was used. The following model is the best QSAR model that has been built.

(M1) $$pIC_{50}=\;-35.7339+3.1806\left(BCUTw-1l\right)+5.2271\left(maxHother\right)-3.5144\left(MDEN-33\right)+0.3912\left(SRW3\right)$$

Both the training and test sets yielded positive results from the constructed model. The four descriptors are shown below along with their statistical characteristics and model contribution. The model 1 descriptors are enough for a dependable QSAR model prediction for freshly developed molecules, as evidenced by the correlation coefficient's (R^2^) proximity to the full number 1.0. The model appears promising, as shown by the result of 0.8086, which indicates that 80.86 % of the residual variation occurs in Table S1. Furthermore, the closeness of Q2loo to internal R2 suggests that the model was not over-fitted. This was shown to be consistent with modeling studies that had already been published (Albratty, 2022).

**(Fitting criteria)**

R^2^ = 0.8086; R^2^_adj_ = 0.79; R^2^-R^2^_adj_ = 0.0187; LOF = 0.1048; Kxx = 0.4387; RMSEtr = 0.2674; MAEtr = 0.188; RSStr = 3.2888; CCCtr = 0.8942; *s* = 0.2832; *F* = 43.3149; Q^2^_loo_ = 0.7559

(**Internal validation criteria**)

Q^2^_loo_ = 0.7559; R^2^-Q^2^_loo_ = 0.0527; RMSEcv = 0.302; MAEcv = 0.212; PRESScv = 4.1947; CCCcv = 0.8657; Q^2^_LMO_ = 0.7358; R^2^_Yscr_ = 0.09; Q^2^_Yscr_ = −0.1518; R^2^_Yrnd_ = 0.0875; Q^2^_Yrnd_ = −0.1552

(**External validation criteria**)

RMSEext = 0.3218; MAEext = 0.262; PRESSext = 1.7609; R^2^ext = 0.7325; Q^2^-F_1_ = 0.6915; Q^2^-F_2_ = 0.6912; Q^2^-F_3_ = 0.7228; CCCext = 0.8536; r2maver. = 0.6263; r2m delta = 0.0815

**Predictions by LOO**:

Exp(x) vs. Pred(y): R^2^ = 0.7571; R'^2^o = 0.702; k' = 0.9981; Clos' = 0.0728; r'2 *m* = 0.5793

Pred(x) vs. Exp(y): R^2^ = 0.7571; R^2^o = 0.756; *k* = 0.9992; Clos = 0.0015; r2m = 0.7316

**External predictions by model equation**:

Exp(x) vs. Pred(y): R^2^ = 0.7325; R'^2^o = 0.7245; k' = 0.9939; Clos' = 0.0109; r'2 *m* = 0.6671

Pred(x) vs. Exp(y): R^2^ = 0.7325; R^2^o = 0.6923; *k* = 1.0032; Clos = 0.0549; r2m = 0.5856

The observed pIC_50_ and validation sets were compared to the anticipated pIC_50_ for the antimalarial modeling parameters in Table S1. The model is error-free, as shown by the computed activities (pIC_50_) values agreeing with the test set (Ugbe et al., 2023). Similarly, further experiments were conducted to guarantee the robustness and dependability of the suggested 2D-QSAR model. First, the cross-correlation matrix was used to evaluate the greatest inter-collinearity among the model's descriptors. The variance inflation factor (VIF), as established by Edache et al. (2016a,b), was then used to evaluate the multi-collinearity of the finished models. The model's descriptors are independent, as shown by the greatest intercorrelation (R^2^) of 0.6373 between any two of the descriptors. Table 3 contains the intercorrelation matrix. Each descriptor's variance inflation factor (VIF) was computed, and none of the results were higher than 5.0, suggesting that the model lacked multi-collinearity. To evaluate each descriptor's relative significance and contribution to the model, the mean effect (MF) value was computed. The MF value shows how important a descriptor is concerning other descriptors in the model, and its sign (+ or -) shows which way the activity values vary as the related descriptor values rise or decrease. Table 3 displays the computed mean effect values. The nhigh lowest atom-weighted BCUTS descriptor has the highest value of 0.9135, while the MDEN-33 descriptor (Molecular distance edge between all tertiary carbons) has the lowest value of −0.0124. Increasing the BCUTw-1l (nhigh lowest atom weighted BCUTS) descriptor by 91.35 % will increase the activity (pIC_50_) of the compounds.

**Table 3:** Descriptors and meaning of descriptors with their matrix, VIF, and MF to the model.

Descriptors	Meaning	BCUTw-1l	maxHother	MDEN-33	SRW3	VIF	MF
BCUTw-1l	nhigh lowest atom weighted BCUTS	1				1.3910	0.9135
maxHother	Maximum atom-type H E-State: H on aaCH, dCH2 or dsCH	−0.22998	1			1.0581	0.0817
MDEN-33	Molecular distance edge between all tertiary carbons	0.302281	−0.05646	1		1.6847	−0.0124
SRW3	Self-returning walk count of order 3 (ln(1 + *x*)	0.493478	−0.07609	0.63725	1	2.0271	0.0173

BCUTw-1l is widely employed in cheminformatics for its ability to capture essential molecular characteristics, making it valuable for predictive modeling and chemical analysis. BCUTw-1l represents the "n-high lowest atom-weighted BCUTS," where the eigenvalues are calculated based on atomic weights placed along the diagonal of the Burden matrix (Bertato et al., 2023). It typically reflects molecular diversity associated with lower eigenvalues weighted by atomic weight. The descriptor increases with higher atomic weights or the presence of specific bonds, such as C–F bonds, which contribute to molecular complexity and diversity (Hook et al., 2013). The maxHother is a 2D electro-topological molecular descriptor that quantifies the maximum atom-type H E-State value for hydrogens in specific bonding environments such as; aaCH (aromatic or aliphatic CH groups), dCH2 (doubly bonded CH₂ groups), and dsCH (doubly bonded sulfur-containing CH groups). The maxHother measures the highest electro-topological state of hydrogens in these environments, which correlates with their ability to participate in weak hydrogen bonding or hydrophobic interactions (Kichev et al., 2023). Higher values indicate hydrogens with greater electron density or polarizability, influencing solubility and molecular reactivity (https://www.vegahub.eu/). This descriptor is particularly valuable for prioritizing molecules with specific hydrogen-bonding capacities in drug discovery and environmental chemistry. The MDEN-33 measures the edge distances between all tertiary nitrogens (nitrogen atoms bonded to three other atoms) in a molecule. This reflects molecular complexity and nitrogen-rich substructures, which are critical in hydrogen bonding, solubility, and bioactivity (Sahu et al., 2023). This descriptor is particularly valuable for prioritizing nitrogen-rich scaffolds in medicinal chemistry and materials science (Toppur and Jaims, 2021). The SRW3 descriptor is not explicitly defined in the provided search results, but based on standard cheminformatics nomenclature and the context of electro-topological state (E-State) descriptors from the Environmental Protection Agency (EPA) guide, it likely represents a weighted molecular descriptor related to structural or electronic features. The SRW3 descriptor prioritize specific atomic properties (e.g., sulfur atoms in thiols, nitrogen lone pairs) for reactivity or toxicity predictions (Alam et al., 2024).

**Fig. 3:**
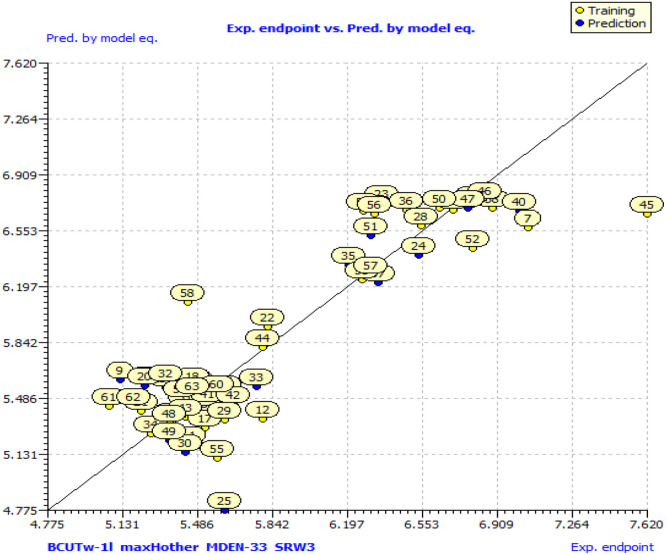
Experimental vs. predicted pIC_50_ values obtained from model 1.

This model has a cross-validated correlation coefficient (Q^2^_loo_) value of >0.5 (0.7559), according to the findings of the fitting requirements. Although Q^2^_loo_ is a significant parameter, it is insufficient to assess the model's power. The true predictive capacity of a QSAR model can only be ascertained via the model validation procedure, which involves forecasting the activities of compounds in the test set (i.e. not included in model formation). The QSAR equation should be rigorously validated to ensure its excellent, dependable prediction and conclusion. This suggests a set of statistical standards and a quantitative assessment. A metric such as R^2^ measures how well experimental results and projected results agree. The model's high quality was further verified with additional statistical tests. For regressions between expected and experimental values through the origin, the correlation coefficient is R’^2^. For regressions between experimental and predicted values via the origin, the correlation coefficient is R_0_’^2^ (Edache et al., 2016a,b). Another measure, R^2^m, was listed as a valuable parameter for the external prediction. The model has an excellent external prediction, as shown by the R^2^m value of 0.8778, which is >0.5 (Edache et al., 2016a,b). The regression lines' slopes (k and k') across the origin have values that are acceptable over the range of 0.85 to 1.15 and are very near to 1. The findings showed that every criterion was met, which gave the constructed model credibility and authority. Fig. 3 shows the predicted pIC_50_ values against their experimental values for both the training and test sets, demonstrating a strong relationship between the four chosen descriptors and the activity of interest. The highest statistical performance was shown by model 1, which captured 80 % of the observed variation. Fitting criteria, internal validation criteria, and external validation criteria are all excellent, and it has the lowest standard deviation and a high F-parameter.

**Fig. 4:**
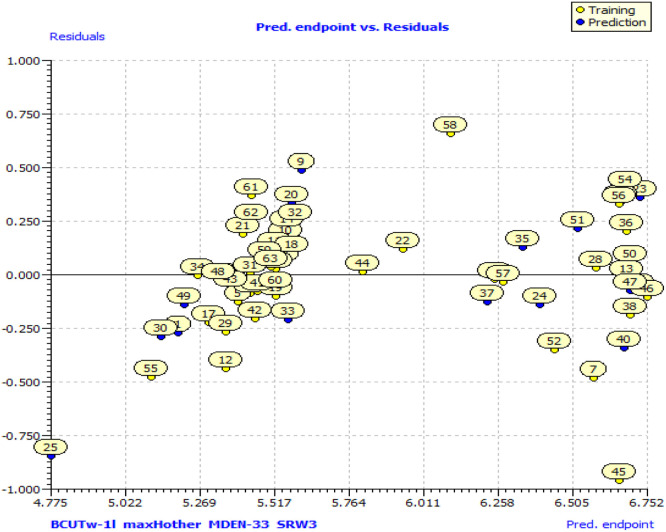
The residuals of 2-aziridinyl- and 2,3-bis(aziridinyl)-1,4-naphthoquinonyl sulfonate and acylate derivatives against the experimental values of pIC50 using the GA-MLR model.

The constructed model was free from systematic error, as shown by the unpredictability of the activities on both the positive and negative sides of the y-axis displayed on the scatter plot between the experimental activity reported in Fig. 4 and the standardized residual activity (Ugbe et al., 2023).

**Fig. 5:**
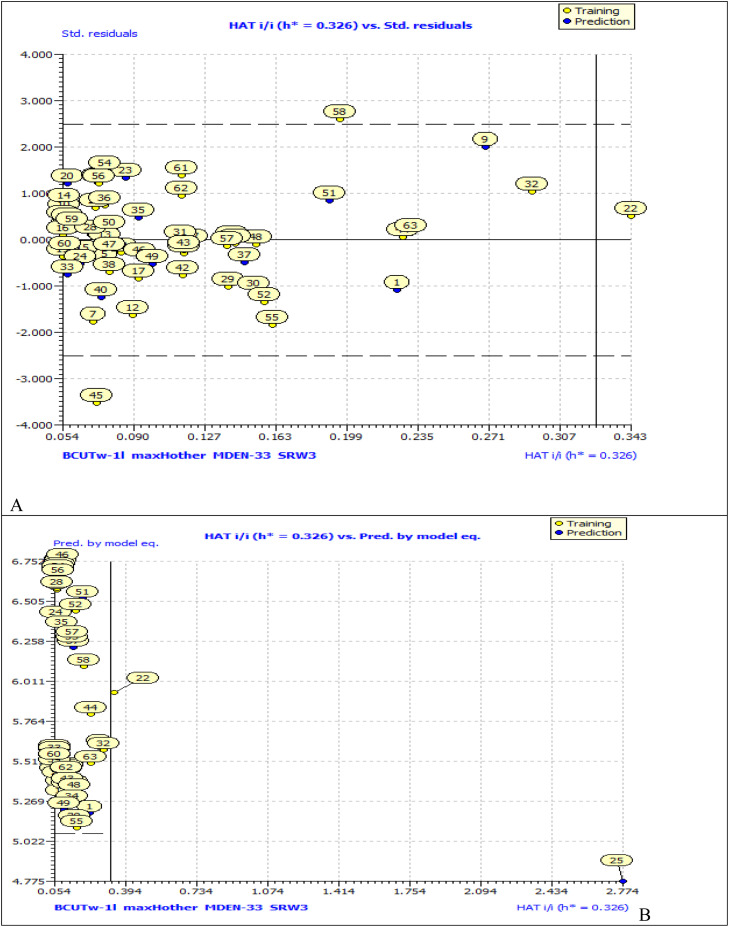
(A) Williams and (B) Insubria plots for residuals versus leverage for model 1.

An important factor in determining the accuracy of pIC_50_ estimates is the assessment of the application domain (AD). It entails figuring out the theoretical geographical area that the model's descriptors specify. Although there isn't one, widely accepted way of determining AD, there are several accepted techniques (Han et al., 2019). In this investigation, the warning leverage, h*, was set at 3p/n (h* = 0.326), where n is the number of training samples and p is the number of descriptors plus one. The square area between the leverage threshold h* of 0.326 and ±2.5 standard deviation was designated as the application domain (AD). All of the compounds in the training and test sets had their leverage values determined (Fig. 5A). By examining the range of each descriptor, the William technique determines which molecules are outside the AD. Only compounds with a leverage value < 0.326 and a standardized residual < ±2.5 standard deviation units were deemed to be within the AD. The graphic indicates that compound 22 of the training set is a structural outlier, meaning it is beyond the designated domain. Compounds 45 and 58 are two inhibitors that exhibit response outliers, with responses above the threshold values of ±2.5 standard deviation units of standardized residual. The structural outlier was kept in the model in compliance with the aforementioned criteria because of its excellent predictability.

To achieve a definitive conclusion in this AD, an Insubria AD method was employed. A molecule is considered outside the applicability domain (AD) if it is classified as such by two or more AD assessment techniques. This Insubria AD approach ensures a comprehensive assessment and enhances the reliability of the AD determination process. The Insubria graph, depicting the diagonal hat elements versus the model equation predictions, is presented in Fig. 5B This graph confirms the applicability domain for externally applied compounds beyond the training set without requiring experimental data. Consideration of this applicability domain is essential for future prediction applications, as each external compound must fall within these boundaries. The model applies to molecules within this defined domain, encompassing not only the structural domain defined by the four model descriptors but also the predicted endpoint value range, which is represented in the Insubria graph rather than the Williams plot. The reliable prediction zone is determined by the critical leverage value (h*) and the minimum and maximum predicted endpoint values from the training set. In this study, all compounds in the external validation set were found to lie within this domain. These show that the forecasts are considered to be accurate estimates and are not based on extrapolation. Fig. 5B displays the Insubria AD plots. Therefore, we can design new compounds with improved values of antimalarial activity using this 2D-QSAR model.

**Fig. 6:**
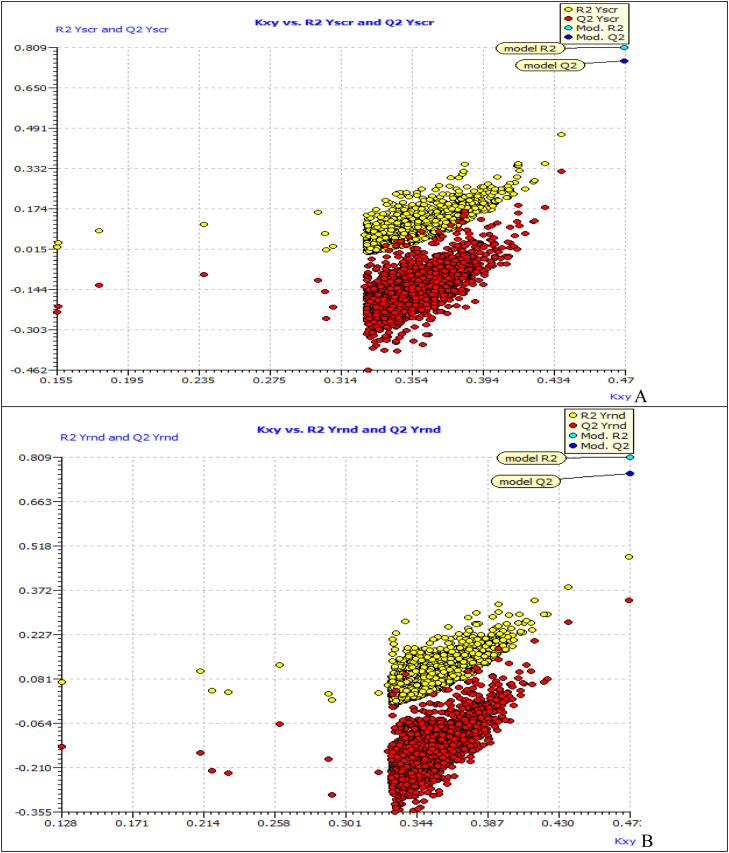
The original model following the application (A) the Y-randomization and (B) Y-scramble methods of model 1.

The model's strength and capacity to forecast the activity of 2-aziridinyl- and 2,3-bis(aziridinyl)-1,4-naphthoquinonyl sulfonate and acylate derivatives against the human malaria parasite *P falciparum* was evaluated using the Y-randomization and Y-scramble techniques. The Q^2^ and R^2^ values for model 1 are substantially lower than the original model following the application of the Y-randomization and Y-scramble methods, as shown in Fig. 6A and 6B This indicates that the suggested model is reliable and may be authorized to forecast the activity of new title inhibitors against the human malaria parasite *P falciparum.*

#### 3.1.1 Validation of the molecular docking protocol

This study used the AutoDock Vina v1.2.5 software to analyze a set of 63 2-aziridinyl- and 2,3-bis(aziridinyl)-1,4-naphthoquinonyl sulfonate and acylate derivatives in-silico to highlight their potential binding affinity and interaction modes with the active site of the human malaria parasite, *P. falciparum*. Table 4 provides a summary of the estimated binding affinity and the estimates of the binding site of the 1MVT enzyme structure for each of the substances under study. According to the molecular docking data, 42 of the 63 compounds exhibited binding affinities that ranged from −9.043 kcal/mol to −11.42 kcal/mol, which was higher than the reference (DTM)'s binding affinity of −9.036 kcal/mol. Compounds 18, 34, and 58 with the highest binding affinities were selected based on the results of docking screening to illustrate the binding manner of the inhibitors of 2-aziridinyl and 2,3-bis(aziridinyl)-1,4-naphthoquinonyl sulfonate and acylate derivatives.

**Table 4:** Estimated binding affinities (kcal/mol) and lipophilicity indices for the 63 disulfides docked with 1MVT.

Job	Affinity	logKi	LE	SILE	FQ	LLE	LELP	Ki
1	−7.305	−5.355	0.562	3.384	0.88	3.343	3.58	4.42 uM
2	−9.961	−7.302	0.356	3.666	0.998	4.414	8.112	49.94 nM
3	−9.841	−7.214	0.339	3.584	0.977	3.564	10.768	61.16 nM
4	−10.18	−7.462	0.351	3.707	1.012	3.921	10.089	34.51 nM
5	−10.3	−7.55	0.278	3.486	0.97	1.292	22.51	28.18 nM
6	−10.35	−7.587	0.334	3.694	1.015	3.774	11.416	25.9 nM
7	−7.58	−5.556	0.474	3.299	0.855	4.647	1.917	2.78 uM
8	−9.221	−6.759	0.307	3.324	0.909	3.309	11.238	174.15 nM
9	−9.455	−6.931	0.305	3.375	0.927	3.612	10.882	117.33 nM
10	−10.08	−7.389	0.348	3.671	1.003	3.897	10.035	40.86 nM
12	−7.362	−5.396	0.387	3.043	0.796	4.906	1.266	4.01 uM
13	−9.82	−7.198	0.378	3.695	0.998	3.583	9.563	63.36 nM
14	−10.09	−7.396	0.348	3.674	1.003	4.2	9.184	40.17 nM
15	−8.371	−6.136	0.322	3.15	0.85	3.678	7.632	731.1 nM
16	−8.318	−6.097	0.362	3.247	0.866	4.639	4.029	799.51 nM
17	−10.29	−7.543	0.294	3.542	0.982	3.436	13.969	28.66 nM
18	−11.42	−8.371	0.357	4.038	1.112	4.33	11.319	4.26 nM
19	−9.178	−6.728	0.316	3.342	0.911	3.699	9.585	187.26 nM
20	−9.756	−7.151	0.325	3.517	0.962	3.651	10.768	70.59 nM
21	−9.008	−6.603	0.273	3.156	0.871	3.379	11.81	249.49 nM
22	−7.501	−5.498	0.441	3.206	0.832	4.199	2.945	3.17 uM
23	−10.18	−7.462	0.351	3.707	1.012	4.02	9.806	34.51 nM
24	−10.61	−7.777	0.332	3.751	1.034	3.251	13.632	16.7 nM
25	−9.897	−7.255	0.291	3.436	0.951	3.504	12.888	55.64 nM
26	−9.023	−6.614	0.376	3.478	0.931	3.246	8.957	243.25 nM
27	−7.314	−5.361	0.43	3.126	0.811	3.487	4.359	4.35 uM
28	−10.83	−7.938	0.319	3.76	1.042	1.261	20.93	11.52 nM
29	−7.275	−5.333	0.52	3.296	0.854	3.616	3.303	4.65 uM
30	−9.333	−6.841	0.322	3.399	0.928	4.829	6.248	144.15 nM
31	−7.166	−5.253	0.478	3.18	0.823	3.988	2.647	5.59 uM
32	−8.783	−6.438	0.338	3.305	0.893	4.842	4.723	364.73 nM
33	−10.19	−7.469	0.364	3.75	1.02	4.812	7.299	33.93 nM
34	−10.81	−7.924	0.349	3.858	1.06	4.072	11.038	11.92 nM
35	−10.13	−7.425	0.405	3.857	1.036	4.694	6.742	37.55 nM
36	−9.698	−7.109	0.373	3.649	0.985	3.198	10.486	77.85 nM
37	−9.686	−7.1	0.404	3.733	1	4.678	5.996	79.45 nM
38	−11.15	−8.173	0.384	4.06	1.107	3.713	11.614	6.71 nM
39	−9.675	−7.092	0.358	3.599	0.974	3.777	9.26	80.93 nM
40	−9.771	−7.162	0.362	3.635	0.985	3.293	10.688	68.83 nM
41	−10.39	−7.616	0.315	3.64	1.005	2.124	17.433	24.21 nM
42	−7.864	−5.764	0.342	3.07	0.818	3.074	7.865	1.72 uM
43	−6.897	−5.056	0.531	3.195	0.832	3.729	2.5	8.8 uM
44	−7.408	−5.43	0.463	3.225	0.835	4.213	2.628	3.71 uM
46	−9.805	−7.187	0.377	3.689	0.996	3.741	9.14	64.99 nM
47	−9.926	−7.276	0.368	3.693	1.002	3.358	10.648	52.98 nM
48	−10.1	−7.403	0.361	3.717	1.012	5.26	5.935	39.5 nM
49	−10.21	−7.484	0.365	3.757	1.023	5.172	6.334	32.81 nM
50	−9.592	−7.031	0.384	3.652	0.983	3.724	8.611	93.1 nM
51	−8.696	−6.374	0.378	3.395	0.905	4.359	5.331	422.42 nM
52	−9.043	−6.629	0.301	3.26	0.891	4.181	8.133	235.18 nM
53	−9.836	−7.21	0.378	3.701	0.998	4.779	6.431	61.68 nM
54	−9.956	−7.298	0.398	3.791	1.018	4.222	7.728	50.37 nM
55	−9.785	−7.173	0.362	3.64	0.985	5.17	5.534	67.22 nM
56	−8.703	−6.379	0.378	3.397	0.905	3.895	6.571	417.46 nM
57	−9.875	−7.238	0.395	3.76	1.011	4.677	6.484	57.75 nM
58	−10.88	−7.975	0.363	3.922	1.075	2.981	13.758	10.59 nM
59	−10.06	−7.374	0.359	3.702	1.006	4.002	9.392	42.26 nM
60	−8.7	−6.377	0.335	3.274	0.885	4.312	6.164	419.58 nM
61	−7.717	−5.657	0.367	3.096	0.817	2.814	7.747	2.2 uM
62	−9.862	−7.229	0.365	3.669	0.993	2.816	12.092	59.03 nM
63	−9.373	−6.871	0.284	3.283	0.906	4.842	7.145	134.74 nM
DTM	−9.036	−6.623	0.335	3.362	0.912	4.459	6.46	237.97 nM

NB: Ligand efficiency (LE); size-independent LE (SILE); fit quality (FQ); Lipophilic ligand efficiency (LLE); ligand efficiency dependent lipophilicity (LELP); estimated inhibition constant (Ki).

All compounds' additional drug-like characteristics, including LE, LLE, FQ, SILE, and LELP values, were anticipated. To improve structural efficiency and turn it from a lead to a therapeutic candidate, lipophilicity is thought to be a fundamental characteristic. For LE > 0.30 kcal/mol/HA, LLE > 0.5 kcal/mol, LELP − 10 < to <10, and MLOGP or LogP < 3, the findings of all compounds' MLOGP or LogP, LE, LLE, and LELP were equivalent to those of standard values. Using the AutoDock Vina method, the chosen compounds and the reference compound (DTM) docked in the active site of the protein targets. The ligand-protein docked poses that were produced were then shown using Discovery Studio.

#### 3.1.2 The binding mode of DTM with the protein target

**Fig. 7:**
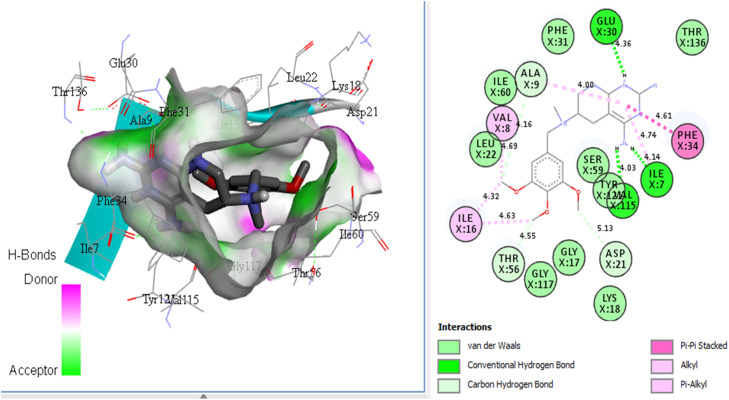
The binding mode interactions of DTM in the pocket site of 1MVT protein target.

One crucial interaction that keeps ligands and receptors stable is the carbon-hydrogen and conventional hydrogen bond. DTM showed that the -NH_2_ and –OCH_3_ moieties formed substantial carbon-hydrogen and conventional hydrogen bonds with the GLU30, ILE7, VAL115, ASP21, THR56, and ALA9 residues in the binding pocket of 1MVT (Fig. 7). The fused ring and –OCH_3_ group with PHE34, ALA9, VAL8, and ILE16 also exhibited a hydrophobic interaction (alkyl, pi-pi stacked, and pi-alkyl) upon closer inspection. A stronger binding affinity may have been provided by the carbon-hydrogen and conventional hydrogen bonds, suggesting dependable stability with protein target active site residues to influence prospective antimalarial activity.

#### 3.1.3 The binding mode of compound 18 with the protein target

**Fig. 8:**
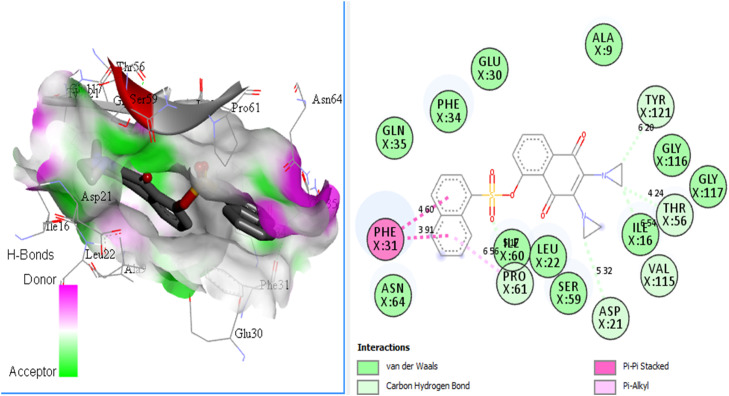
The binding mode interactions of compound 18 in the pocket site of 1MVT protein target.

Regarding compound 18′s binding mechanism, its -NCH_2_- groups formed carbon-hydrogen bonds with TYR121, THR56, ILE16, and ASP21. The fused ring, which had invested in PHE31 and PRO61 via pi-alkyl and pi-pi stacking interactions, provided further stability. Potentially substantial inhibitory activity against the malaria parasite is encouraged by these crucial interactions that provide stability (Fig. 8).

#### 3.1.4 The binding mode of compound 38 with the protein target

**Fig. 9:**
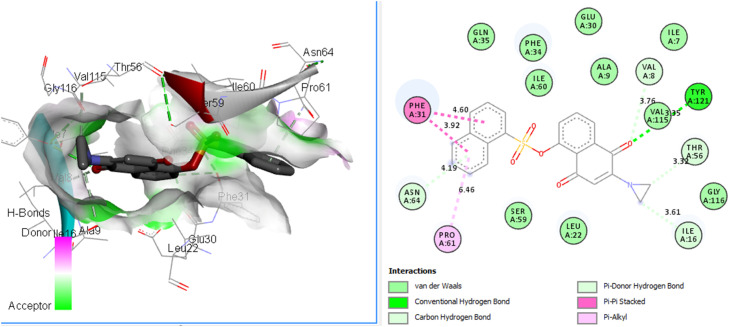
Binding mode interactions of compound 38 in the pocket site of 1MVT protein target.

The fused ring, -CO-, and NH_2_ groups, on the other hand, formed carbon-hydrogen, conventional hydrogen, and pi-donor hydrogen bonds with the amino acid residues VAL8, TYR121, THR56, ILE16, and ASN64 in the binding cavity of 1MVT, while the fused ring provided stability by interacting hydrophobically (pi-alkyl) with PHE31 and PRO61 (Fig. 9).

#### 3.1.5 The binding mode of compound 58 with the protein target

**Fig. 10:**
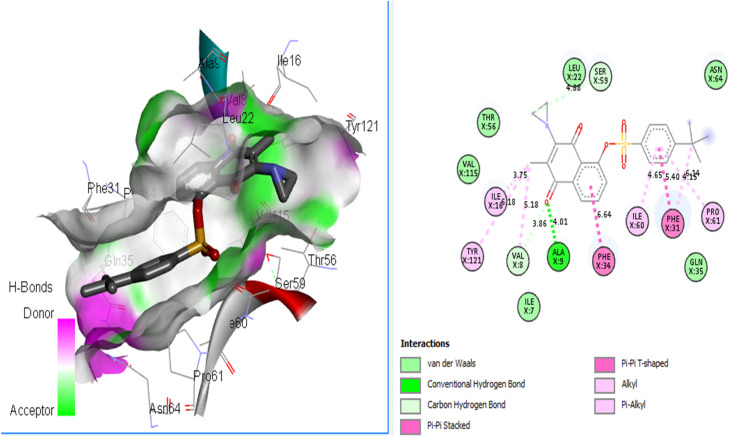
Binding mode interactions of compound 58 in the pocket site of 1MVT protein target.

While the -CO and -NH_2_ groups of compounds 58 approached VAL8 and LEU22 in a carbon-hydrogen bond, the -CO group formed a typical hydrogen connection with ALA9. By investing in ILE60, PHE32, PRO61, PHE34, VAL8, ILE16, and TYR121 via hydrophobic contacts (i.e., pi-pi stacked, pi-pi T-shape, pi-alkyl, and alkyl), the fused ring, phenyl ring, and methyl group provided further stability. Potentially substantial inhibitory activity against the malaria parasite is encouraged by these crucial interactions that provide stability (Fig. 10).

#### 3.1.6 Protein analysis based on pharmacophore features

The 40 most important targets for the typical DTM (reference) compound have been revealed via pharmacophore mapping using the Pharmit web service. Fig. 2 shows the pharmacophore mapping of typical DTM to the active sites of the target protein. A protein's three-dimensional structure is essential for facilitating potential therapeutic interactions with biological activity and for forecasting the likelihood of effectiveness before production. Six chemical characteristics in all were noted, comprising two hydrophobic groups, one positive ion, one aromatic, one H-bond acceptor, and one H-bond donor in addition (Fig. 2).

#### 3.1.7 Validation of the docking-based pharmacophore modeling

However, in terms of drug discovery research, only the potential targets were chosen. Docking affinity scores were used to filter the 40 targets. Fig. S1 lists all of the target compounds that were found while Table 5 lists their docking affinity and calculated metric ratings of the compounds.

**Table 5:** The pharmacophore compounds and their binding affinity towards the protein.

Ligand	Affinity	logKi	LE	SILE	FQ	LLE	LELP	Ki
Pharm1	−8.398	−6.156	0.4	3.369	0.89	4.933	3.058	698.53 nM
Pharm2	−8.479	−6.215	0.404	3.402	0.899	4.992	3.027	609.27 nM
Pharm3	−8.969	−6.574	0.169	2.726	0.767	0.636	35.137	266.46 nM
Pharm4	−4.788	−3.51	0.094	1.472	0.415	−5.979	100.947	309.28 uM
Pharm5	−6.241	−4.575	0.12	1.907	0.537	−1.393	49.733	26.62 uM
Pharm6	−11.5	−8.43	0.24	3.6	1.016	2.852	23.243	3.72 nM
Pharm7	−9.055	−6.637	0.131	2.542	0.708	−4.163	82.44	230.46 nM
Pharm8	−8.902	−6.525	0.27	3.118	0.862	1.782	17.565	298.36 nM
Pharm9	−9.028	−6.618	0.311	3.288	0.896	3.18	11.055	241.21 nM
Pharm10	−9.871	−7.236	0.253	3.289	0.918	3.384	15.223	58.14 nM
Pharm11	−10.98	−8.048	0.282	3.658	1.024	2.039	21.31	8.94 nM
Pharm12	−10.69	−7.836	0.281	3.59	1	3.524	15.345	14.59 nM
Pharm13	−8.617	−6.316	0.278	3.076	0.845	0.785	19.896	482.68 nM
Pharm14	−8.451	−6.195	0.313	3.144	0.852	2.423	12.052	638.76 nM
Pharm15	−10.66	−7.814	0.237	3.402	0.957	2.305	23.244	15.35 nM
Pharm16	−10.13	−7.425	0.267	3.402	0.95	2.924	16.857	37.55 nM
Pharm17	−8.209	−6.017	0.328	3.125	0.839	3.105	8.877	961.0 nM
Pharm18	−10.73	−7.865	0.307	3.693	1.026	2.341	17.993	13.64 nM
Pharm19	−9.33	−6.839	0.17	2.804	0.791	0.13	39.463	144.88 nM
Pharm20	−7.929	−5.812	0.106	2.171	0.603	−6.718	118.207	1.54 uM
Pharm21	−10.99	−8.056	0.282	3.662	1.024	5.524	8.978	8.79 nM
Pharm22	−3.563	−2.612	0.035	0.892	0.236	−24.939	787.18	2.45 mM
Pharm23	−5.257	−3.853	0.146	1.794	0.499	2.299	10.644	140.14 uM
Pharm24	−5.214	−3.822	0.145	1.779	0.495	2.268	10.717	150.69 uM
Pharm25	−10.54	−7.726	0.285	3.568	0.994	2.118	19.677	18.8 nM
Pharm26	−11.1	−8.136	0.277	3.67	1.025	1.828	22.771	7.3 nM
Pharm27	−8.613	−6.313	0.261	3.017	0.833	4.352	7.514	485.95 nM
Pharm28	−7.721	−5.66	0.209	2.613	0.729	2.23	16.411	2.19 uM
Pharm29	−8.498	−6.229	0.327	3.198	0.864	2.567	11.2	590.04 nM
Pharm30	−9.978	−7.314	0.344	3.633	0.991	3.556	10.925	48.53 nM
Pharm31	−8.692	−6.371	0.223	2.896	0.81	0.945	24.332	425.28 nM
Pharm32	−7.635	−5.597	0.402	3.156	0.826	2.216	8.411	2.53 uM
Pharm33	−9.123	−6.687	0.326	3.357	0.914	3.59	9.501	205.47 nM
Pharm34	−9.189	−6.736	0.133	2.58	0.719	−4.288	82.884	183.81 nM
Pharm35	−10.54	−7.726	0.257	3.459	0.969	2.696	19.572	18.8 nM
Pharm36	−7.816	−5.729	0.34	3.051	0.814	1.561	12.258	1.87 uM
Pharm37	−9.881	−7.243	0.282	3.401	0.942	2.726	16.018	57.17 nM
Pharm38	−9.76	−7.154	0.264	3.304	0.921	2.9	16.112	70.12 nM
Pharm39	−8.956	−6.565	0.213	2.918	0.818	1.926	21.778	272.37 nM
Pharm40	−10.52	−7.711	0.309	3.652	1.009	4.996	8.787	19.44 nM

**Fig. 11:**
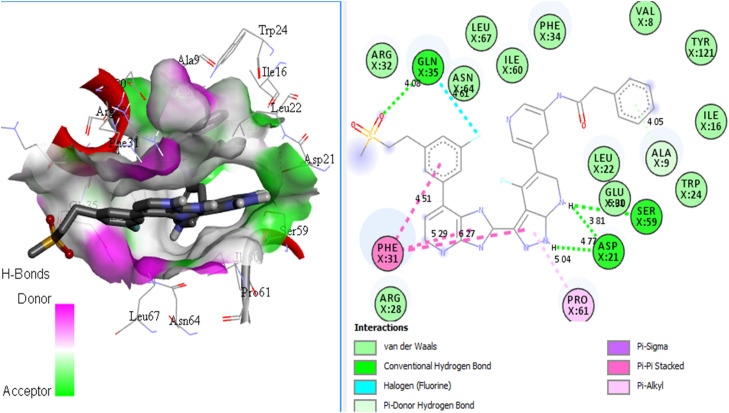
The binding mode interactions of pharm6 in the pocket site of 1MVT protein target.

**Fig. 12:**
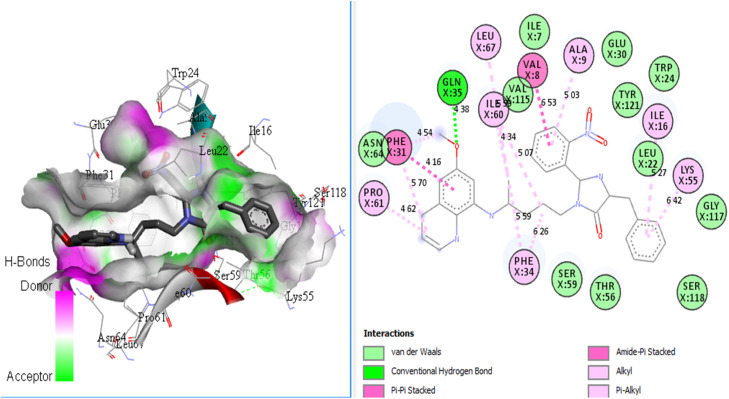
The binding mode interactions of pharm26 in the pocket site of 1MVT protein target.

Correct binding modes and excellent binding affinities were shown by Pharm6 out of the 40 compounds that were evaluated. The following are the potential compounds: Pharm26, Pharm21, Pharm11, Pharm18, Pharm12, Pharm15, Pharm25, Pharm35, Pharm40, Pharm16, Pharm30, Pharm37, Pharm10, Pharm38, Pharm19, Pharm34, Pharm33, and Pharm7, respectively. As representative structures, the most ideal modes and the binding poses of the top two compounds with the greatest binding affinity ratings were chosen for an in-depth examination. The co-crystallized ligand (DTM) within the active malaria receptor (PDB ID: 1MVT) was first examined for binding interactions and orientation. The results showed a binding affinity value of −9.036 kcal/mol as stated above (Table 4). It demonstrated a distinctive three-hydrogen bonding and three-carbon-hydrogen bonding interaction with the important amino acids GLU30, ILE7, VAL115, ASP21, THR56, and ALA9, respectively, via -NH_2_ and –OCH_3_. Furthermore, the amino acid residues PHE34, VAL8, ILE16, ALA9, and ILE7 established six hydrophobic contacts with the fused ring and –OCH_3_, as seen in Fig. 7. Docking study findings demonstrated that the pharmacophore ligands' orientations and binding interactions resembled those of DMT against the malaria receptor. The most influential pharmacophore modeling drug-like compounds, Pharm6 and Pharm26, have binding modes within the target protein's active site that are shown in both three and two dimensions in Fig. 11, Fig. 12. Pharm6 demonstrated an intriguing binding mechanism against the malaria receptor with a binding affinity score of −11.5 kcal/mol, which was comparable to that of the reference ligand. It maintains the traditional hydrogen bonding connections (Pi-donor hydrogen bond) with the important amino acids ASP21 and ALA9. Two more typical hydrogen bonds were also established with the GLN35 and SER59 residues. Additionally, four hydrophobic interactions with amino acid residues PHE31 and PRO61 and one halogen (fluorine) contact with GLN35 were integrated with Pharm6 (Fig. 11).

The binding affinity for Pharm26 was −11.1 kcal/mol. Through a single, typical hydrogen bonding contact with the GLN35 residue, this ligand demonstrated the second-best binding mechanism into the target protein, totally occupying the protein. Furthermore, the Pharm26 molecule's whole structure established eleven hydrophobic contacts with the residues of PRO61, PHE31, LEU67, ILE60, ALA9, VAL8, ILE16, LYS55, and PHE34 (Fig. 12).

#### 3.1.8 ADME/Tox prediction and bioavailability

The online servers SwissADME and PKCSM were used to estimate the synthetic accessibility, bioavailability, and Lipinski's rule of the chosen compounds and the control molecule (DTM). We assessed several physicochemical characteristics of bioavailability (Table 6) and some pharmacokinetic features (ADME/Tox) shown in Table 7 to make sure the chosen drug-like compounds are viable medications and have synthetic accessibility in comparison to the control. Molecular weight (WM), the number of hydrogen bond donors (HBD), the number of hydrogen bond acceptors (HBA), the number of rotatable bonds (NRB), and the MLOGP are the five physicochemical factors that make Lipinski's rule (Lipinski et al., 1997). The values derived from these parameters are compiled in Table 6. One of the most crucial molecular characteristics is MLOGP, which has a big impact on a lot of ADMET-related factors as well as the general quality of the drug. In terms of MLOGP values, there is a definite trend, with the more promising medications exhibiting lower MLOGP values. Given that these particular drug-like compounds must penetrate the malaria cell wall to reach their intended target, this may be a crucial factor to take into account. All of the chosen compounds, except pharm6 and pharm26, have molecular weights under 500 g/mol. For the molecule to penetrate and pass through the lipid bilayer membrane, hydrogen bonds must be disrupted, which increases solubility in water. Therefore, for passive diffusion penetration, a greater number of hydrogen bonds decreases partitioning from the aqueous phase into the lipid bilayer membrane (Shinoda, 2016). The screened drug-like compounds have many hydrogen bonds at the proper Lipinski interval. Furthermore, every chosen drug-like compound, except pharm6, has TPSA values between 66.46 and 121.64 Å^2^, allowing them to exhibit excellent correlation with human intestinal absorption, the permeability of the Caco-2 monolayer, and the penetration of the blood-brain barrier (Edache et al., 2024c). Except for pharm26, all of the chosen compounds are probably the most flexible, with multiple rotatable bonds of 10. According to Table 6 findings, drug-like compounds 18, 34, and 58 generally meet Lipinski and Veber's requirements, meaning they won't have any issues with oral bioavailability. When choosing a medication, synthetic accessibility (SA) is thought to be the most important consideration. The main premise behind the SA score is that the synthesis capability is correlated with the frequency of molecular fragment's inaccessible molecules. From 1 (extremely easy) to 10 (very tough), SA scores fall within this range. The compound with the highest SA of 6.01 was compound 34. Compound 18 also received a respectable value of 3.92. The drug-like compounds 18 and 58 were found to have greater values in the SA filter when compared to the control compound DTM (SA = 2.93).

**Table 6:** Lipinski rule, bioavailability score, and synthetic accessibility results of the compounds.

Number	MW g/mol	HBA	HBD	MLOGP	TPSA	NBR	Bioavailability score	Synthetic accessibility
	< 500	< 10	< 5	< 4.15	< 140	< 10	Higher is better	Lower is better
DTM	370.41	6	2	−0.24	121.64	6	0.55	2.93
18	446.48	5	0	1.77	91.91	5	0.55	3.92
34	418.48	4	0	2.17	66.46	5	0.55	6.01
58	425.5	5	0	2.53	88.9	5	0.55	3.94
Pharm6	664.68	10	3	3.2	167.65	10	0.17	4.36
Pharm26	539.62	6	2	2.46	112.31	11	0.55	4.71

**Table 7:** ADMET results predicted in silico of the selected compounds.

	Absorption	Distribution		Metabolism					Excretion		Toxicity		
	GI absorption	BBB permeant	Pgp substrate	CYP1A2 inhibitor	CYP2C19 inhibitor	CYP2C9 inhibitor	CYP2D6 inhibitor	CYP3A4 inhibitor	Total Clearance	Renal OCT2 substrate	AMES toxicity	hERG I inhibitor	Skin Sensitisation
DTM	High	No	Yes	No	No	Yes	Yes	Yes	0.29	No	No	No	No
18	High	No	No	Yes	Yes	Yes	No	Yes	0.657	No	No	No	No
34	High	No	No	No	Yes	Yes	Yes	Yes	0.258	No	No	No	No
58	High	No	No	No	Yes	Yes	No	Yes	0.416	No	No	No	No
Pharm6	Low	No	No	No	No	Yes	No	Yes	0.577	No	No	No	No
Pharm26	High	No	Yes	Yes	Yes	Yes	Yes	Yes	1.022	Yes	Yes	No	No

A thorough grasp of toxicology and pharmacology is essential to the advancement of therapeutic drug-like development. Gaining this understanding improves the success rate in addition to shortening the time needed to produce a drug. The online web server is used to retrieve the parameters for the chosen drug-like compounds. All of the studied drug-like compounds have been shown to have good solubility in the gastrointestinal (GI) environment using Swiss ADME calculations. Furthermore, Table 7 shows that there is no proof that these drug-like substances meet the requirements needed to cross the blood-brain barrier (BBB), indicating that they are unable to enter the central nervous system. Their potential for absorption, permeability, and retention inside the body is increased by the noteworthy discovery that these drug-like compounds are not P-glycoprotein inhibitors when compared to the control compound (DTM). By providing insightful information about the possible interactions of these drug-like substances with cytochrome enzymes, namely the CYP450 family, SwissADME analysis broadens its applicability. These enzymes play a critical role in pharmacology and drug development since they are necessary for drug metabolism (Edache et al., 2024a). Making educated judgments throughout the drug development process requires an understanding of how a medication may affect or be impacted by these crucial enzymes, as shown in Table 7.

#### 3.1.9 Validation of the MD simulations protocol

**Fig. 13:**
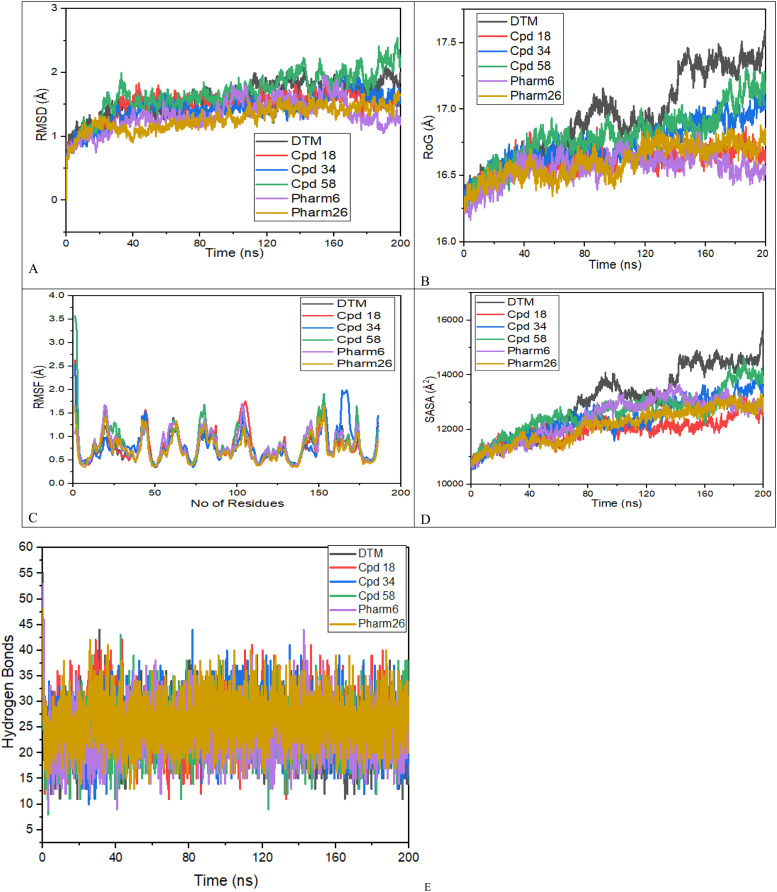
Molecular dynamic simulations result in over 200 ns of simulation, (A) RMSD, (B) RoG, (C)RMSF, (D) SASA, and (E) H-bonds.

To assess the stability of the protein-ligand complexes, the RMSD of their backbone atoms was examined. Two significant trajectories ligand mobility and ligand conformation were examined during MD simulation investigations and reported in terms of RMSD in Å. RMSD values < 3.0 Å were found for every protein-ligand combination that was examined. The compound 18, compound 32, Pharm6, and Pharm26 complexes exhibit the lowest RMSD compared to the standard (DTM-complex), as shown in Table 8 and Fig. 13A. Remarkably, the compound 58 complex's RMSD is rather greater than the DTM complex's, indicating the standard's notable stability. These values (> 3 Å) indicate that the ligands in each protein cavity have reoriented. The ligands are well inside the active site cavity, according to structural analysis. All five compounds are stable, according to the RMSD plot, which shows no unanticipated increases in RMSD values throughout the simulation (Fig 13A).

**Table 8:** The molecular dynamics simulations for all the selected compounds including the control.

Parameter	DTM	Cpd 18	Cpd 34	Cpd 58	Pharm6	Pharm26
RMSD	1.593	1.488	1.442	1.693	1.335	1.287
RoG	16.937	16.619	16.729	16.796	16.561	16.606
RMSF	0.769	0.787	0.808	0.869	0.813	0.740
SASA	13,071.039	11,982.184	12,391.868	12,644.959	12,424.502	12,153.261
HBond	24.507	25.789	24.968	24.036	23.903	26.111

NB: All parameters’ values are in angstroms (Å) except the SASA value is angstrom square (Å^2^).

We may evaluate the changes in compactness of a ligand-protein complex using the radius of gyration (RoG). In summary, a higher RoG reduces the ligand-protein complex's compactness. During the MD simulation, RoG is employed to ascertain if the complexes are unfolded or folded steadily. The reference compound's (DTM) average RoG value was determined to be 16.937 Å. Additionally, the drug-like compounds 18, 34, 58, pharm6, and pharm26 complexes had average RoG values of 16.619, 16.561, 16.729, 16.796, and 16.606 correspondingly, all of which were much lower than the control molecule (Table 8). As previously mentioned, the DTM-protein complex may be viewed as stably folded if its RoG value remains generally constant during the MD simulation; if it changes over time, it would be regarded as unfolded (Okumura, 2012). Consequently, it can be shown that in comparison to the reference (Fig. 13B), each complex had consistent values of RoG and very comparable compactness behavior. It shows that they have outstanding stability and are perfectly layered on one another.

The flexibility of each residue is assessed to better understand how ligand binding affects receptor flexibility since RMSF helps to identify the area of the receptor that varies over simulation. Fig. 13C illustrates that, in contrast to all other complexes, the binding of pharm26 renders the receptor the most flexible in almost every location. For DTM, drug-like compounds 18, 34, 58, pharm6, and pharm26, the mean RMSF values were determined to be 0.769 Å, 0.787 Å, 0.808 Å, 0.869 Å, 0.813 Å, and 0.740 Å, respectively. For each of the six complexes, the fluctuation pattern was comparable. The residues 1–2, 42–45, 60–64, 77–81, 84–85, 102–104, 150–154, 164–168, and 186 showed notable peaks. According to the RMSF study, compound 18 and pharm26, two of the top six lead drug-like compounds, had the highest stability with protein when compared to the control (Table 8). The RMSF investigation showed that the complexes exhibited fewer variations in the permitted range (Fig. 13C).

A greater SASA score indicates that the protein volume is increasing with time, less variability is generally anticipated. Any drug-like compound may change SASA, and this can significantly affect the structure of the receptor. During the simulation period, the SASA values of all the complexes, including the control, were found to be the lowest, indicating that the presence of these molecules may be able to restrict the growth of proteins (Fig. 13D). The complexes' volume did not significantly increase, according to the SASA values (Table 8). Table 8 shows that drug-like compound 18 has the lowest SASA value, indicating that the protein remains compact over the simulation period and the ligand remains stable within the target protein's binding pocket. The total number of hydrogen bonds in the protein-ligand complexes was ascertained since intermolecular hydrogen bonding is known to support conformational stability. Over the course of the simulation, the Pharm6 complex has the fewest hydrogen bonds, whereas the pharm26–1MVT complex has the most (Fig. 13E). In contrast to the DTM complex, the other complexes have a significant amount of hydrogen bonds. All of the complexes showed a significant number of hydrogen bonds throughout the simulation, which further clarified their conformational stability (Fig. 13E). Additionally, the stability of these five drug-like compounds with the 1MVT receptor was reaffirmed by MD simulation data.

#### 3.1.10 Linear interaction energy (LIE) analysis

Investigating the ligand binding energy of the chosen drug-like compounds is the goal of the LIE estimation implementation. One hundred frames of each protein-ligand MD trajectory were utilized to determine the LIE binding energy. According to the LIE estimate, the final interaction energy for DTM, drug-like compounds 18, 34, 58, pharm6, and pharm26 was −5.8453, −6.1237, −9.4075, 127.2158, 2.6975, and −19.2614 kcal/mol, respectively. Table 9 displays the specific LIE phrases. The exergonic binding process, which is thermodynamically advantageous, is shown by negative values. The positive values are unfavorable under the parameters taken into consideration and point to an endergonic binding mechanism (Lämmerhofer, 2010). More negative numbers correspond to stronger binding. In comparison to the reference compound (DTM), drug-like compounds 18, 34, and pharm26 were shown to have the greatest linear interaction energy.

**Table 9:** The LIE energy estimation of the malaria inhibitor complexes.

Linear interaction energy (LIE)						
Parameters	DTM	Cpd 18	Cpd 34	Cpd 58	Pharm6	Pharm26
Vdw:	−28.2465	−32.0124	−37.9243	731.5299	−32.9597	−51.2342
Elec:	−2.3058	−1.0954	−7.8216	−13.5139	26.1524	−30.4219
**Total:**	**−5.8453**	**−6.1237**	**−9.4075**	**127.2158**	**2.6975**	**−19.2614**

NB: Final: (alpha0.18, beta = 0.33, gamma = 0.0); All energy values are in kcal/mol.

#### 3.1.11 Post-MD simulations binding free energy results

The binding free energies of the simulated complexes were computed using the MM/ PB(GB)SA methodology to get more understanding of the molecular interactions among the chosen drug-like compounds, *Plasmodium falciparum* active site residues, and malaria parasites. The binding free energy of the previous 100 frames was calculated from MD trajectories using the MM/PBSA technique. Except for drug-like compound 58 and the reference compound (DTM), all of the complexes had negative total binding energies, indicating higher binding (Table 10). The drug-like compound's most stable shape was indicated by the compound 18 complex, which had the lowest binding free energy (−17.4324 kcal/mol). Except for the reference compound (DTM) and compound 58, which suggested an endergonic binding mechanism that was unfavorable under the circumstances taken into consideration and less likely to form a stable complex, the other complexes also had low binding energies (exergonic binding process), indicating that they may be used as possible drug-like compounds. Table 10 provides specifics on how each energy parameter contributes to the net free binding energy values.

**Table 10:** The MM/PBSA values of the ligand complexes (kcal/mol).

Parameter	DTM (Mean/SD)	Cpd 18 (Mean/SD)	Cpd 34 (Mean/SD)	Cpd 58 (Mean/SD)	Pharm6 (Mean/SD)	Pharm26 (Mean/SD)
Elec:	−2.3058/11.2546	−1.0954/2.6106	−7.8216/3.2209	−13.5139/5.6246	26.1523/6.6519	−30.4219/5.6816
Vdw:	−28.2465/2.6210	−32.0124/1.8074	−37.9243/1.9993	731.5299/20.3886	−32.9597/2.4075	−51.2341/2.2503
PB:	39.8949/10.5344	20.5522/3.2980	40.7291/3.8385	48.8873/4.9211	8.9285/6.4379	78.3279/6.7363
SA:	−4.7999/0.1267	−4.8768/0.1720	−5.4520/0.1334	−5.6746/0.0974	−5.3269/0.1707	−6.6849/0.1134
Gas:	−30.5523/11.0416	−33.1078/3.2294	−45.7460/3.4345	718.0160/20.2909	−6.8073/6.4797	−81.6560/5.9402
Sol:	35.0950/10.4386	15.6754/3.2387	35.2772/3.8004	43.2126/4.9070	3.6016/6.3714	71.6431/6.7412
Pol:	37.5891/3.6967	19.4568/2.3831	32.9075/2.7479	35.3733/2.5564	35.0809/3.8808	47.9061/3.4622
Npol:	−33.0463/2.6377	−36.8892/1.8625	−43.3763/2.0394	725.8553/20.3843	−38.2866/2.4976	−57.9190/2.2903
**Total:**	**4.5427/3.1024**	**−17.4324/2.2743**	**−10.4688/2.7847**	**761.2286/20.3891**	**−3.2057/3.1403**	**−10.0129/3.7152**

NB: All energy values are in kcal/mol; Cpd = Compound.

Using the MM/GBSA methodology, more research was done to forecast the free binding energies of the protein-ligand simulation complexes (Edache et al., 2024a). In comparison to the conventional DTM complex, which has a binding energy of −13.087 kcal/mol, pharm26 had the greatest binding free energy (−30.822 kcal/mol), followed by compound 34 (−25.8649 kcal/mol) and compound 18 complex (−21.2 kcal/mol), according to the MM/GBSA projected free energies. With bind-free energy values of 736.2326 and −12.5584 kcal/mol, respectively, Compound 58 (endergonic binding) got the lowest value while pharm6 (exergonic binding process) came in second. Excellent stable complexes are indicated by negative net binding free energy, which is a result of the parameters determined from the values of the complexes' binding free energies using the MM/GBSA free energy against the binding mechanism acquired from the molecular dynamic simulations (Table 11). These drug-like compounds 18, 36, and pharm26 have free binding energy values that are higher than the reference molecule (DTM). This might be because, in molecular dynamics simulation studies, numerous complex dynamics conformations are chosen rather than simply one simulated conformation. Additionally, it shows that the compounds' binding free energy increases as the simulation goes on. Table 11 provides specifics on how each energy parameter contributes to the net binding energy values.

**Table 11:** The calculated MM/GBSA binding energies for the complexes and the reference molecule DTM.

Parameter	DTM	Cpd 18	Cpd 34	Cpd 58	Pharm6	Pharm26
△E(internal):	0	−0	−0	−0	0	−0
△E(electrostatic)+ △G(sol):	19.2176	12.3335	13.0571		11.1843	22.8733
△E(VDW):	−32.3046	−33.5335	−38.922	9.7096	−23.7427	−53.6953
**△G binding:**	**−13.087 ± 0.0822 (kcal/mol)**	**−21.2 ± 0.0661 (kcal/mol)**	**−25.8649 ± 0.065 (kcal/mol)**	**736.2326 ± 0.5264 (kcal/mol)**	**−12.5584 ± 0.1001 (kcal/mol)**	**−30.822 ± 0.1562 (kcal/mol)**

NB; Cpd = Compound.

## 4 Conclusions

This work helped to build mathematical models that could quantify the correlation between the molecular structure of a series of 2-aziridinyl- and 2,3-bis(aziridinyl)-1,4-naphthoquinonyl sulfonate and acylate derivatives and their biological antimalarial activity. The molecular characteristics required for strong action to suppress *Plasmodium falciparum* symptoms and the malaria parasite, and so produce an antimalarial response, were also identified in this study. Through internal and external validations, a Y-randomization test, and an application domain, the resultant QSAR models were examined and verified for statistical significance and predictive capability. The analysis's QSAR models' predictive power has shown that the suggested model 1 is very effective using this approach. The following four significant descriptors have an impact on the biological inhibitory action of *Plasmodium falciparum*, the malaria parasite, according to the study of the constructed QSAR model equations. The best candidate for inhibiting the human malaria parasite, *P. falciparum*, is compounds 18, 34, and 54, according to a molecular docking analysis conducted for the derivatives with the *P. falciparum* receptor. Additionally, molecular docking results demonstrated that compounds 18, 34, and 54 binding affinities in the human malaria parasite *P. falciparum* receptor pocket are greater than the control compound's binding affinity with the same receptor, suggesting that compounds 18, 34, and 54 and the human malaria parasite *P. falciparum* receptor are well stable. Furthermore, the in silico pharmacokinetic assessment of ADMET features between the chosen compounds and the control suggests that drug-like compound 18 has superior pharmacokinetic characteristics. Metrics calculated for compound 18 were greater than those calculated for the control DTM, which may be explained by this. By preventing the *P. falciparum* protein's enzymatic activity, compound 18 may thus be suggested as a potential therapy for malaria. Free binding energies and molecular dynamics simulations also showed that drug-like compound 18 occupied the receptor's binding site and stayed there for 200 ns. Since stable bond interactions with amino acids in the binding site were noted, this is supported by RMSD, RoG, RMSF, SASA, and H-bonds analysis. The facts and insights presented in this paper may provide medicinal chemists with several options to create new antimalarial medications by using compound 18′s structure to create new compounds with more potent biological activity. Therefore, 2-aziridinyl- and 2,3-bis(aziridinyl)-1,4-naphthoquinonyl sulfonate and acylate derivatives' structures might be a potential avenue for the creation of novel medications to treat malaria.

In subsequent research, we will create hologram-QSAR models based on the series of 2-aziridinyl- and 2,3-bis(aziridinyl)-1,4-naphthoquinonyl sulfonate and acylate derivatives. To propose new derivatives of 2-aziridinyl- and 2,3-bis(aziridinyl)-1,4-naphthoquinonyl sulfonate and acylate structure, we will use compound 18 as a reference compound in the design of novel inhibitors of the human malaria parasite, P. falciparum enzymatic activity. Additionally, we will utilize compound 18 as a model of malaria treatment.

## Funding

This research received no external funding.

## Author contributions

E.I.E: Conceptualisation, Formal analysis, Investigation, Writing-Original Draft. A.A and A.U: Formal analysis, Methodology, Writing- Original Draft. H.A.D: Supervision, Writing – review & editing. H.A.D, A.A, and F.A.U: Supervision, Writing – review & editing. E.I.E and F.A.U: Conceptualisation, Resources, Supervision, Writing – review & editing. All authors reviewed the manuscript.

## Declaration of competing interest

The authors declare that they have no known competing financial interests or personal relationships that could have appeared to influence the work reported in this paper.

## Data Availability

Data will be made available on request.

## Appendix Supplementary materials

Image, application 1
