# Supplementary material for: Pharmacophore modeling, 2D-QSAR, drug likeness and toxicity prediction of 2-aziridinyl- and 2,3-bis(aziridinyl)-1,4-naphthoquinonyl sulfonate and acylate derivatives as multifunctional agents for the treatment of malaria parasite, Plasmodium falciparum
Source: NAM J. 2025 Apr 17;1:100019. doi: 10.1016/j.namjnl.2025.100019 (PMC13289209; doi:10.1016/j.namjnl.2025.100019)
Supplement: Supplementary file 1 [file mmc1.docx]

Table S1. Experimental data pIC50 and predicted values with the residue values of molecules of the present dataset.

| Name | Status | Exp. pIC50 | Predicted pIC50 | Residues |
| --- | --- | --- | --- | --- |
| 1 | Prediction | 5.4685 | 5.1981 | -0.2704 |
| 2 | Prediction | 5.2366 | 5.5736 | 0.337 |
| 3 | Training | 5.4318 | 5.5545 | 0.1227 |
| 4 | Training | 5.4949 | 5.5559 | 0.061 |
| 5 | Training | 5.5229 | 5.3962 | -0.1267 |
| 6 | Prediction | 5.4089 | 5.5041 | 0.0952 |
| 7 | Training | 7.0555 | 6.5752 | -0.4803 |
| 8 | Training | 5.3979 | 5.5444 | 0.1465 |
| 9 | Prediction | 5.1192 | 5.607 | 0.4878 |
| 10 | Training | 5.3872 | 5.5553 | 0.1681 |
| 11 | Training | 5.3979 | 5.5182 | 0.1203 |
| 12 | Training | 5.7959 | 5.3583 | -0.4376 |
| 13 | Training | 6.699 | 6.6874 | -0.0116 |
| 14 | Training | 5.3372 | 5.5575 | 0.2203 |
| 15 | Training | 5.5229 | 5.436 | -0.0869 |
| 16 | Training | 5.4949 | 5.5215 | 0.0266 |
| 17 | Training | 5.5229 | 5.2993 | -0.2236 |
| 18 | Training | 5.4685 | 5.5736 | 0.1051 |
| 19 | Training | 5.6198 | 5.5214 | -0.0984 |
| 20 | Prediction | 5.2366 | 5.5736 | 0.337 |
| 21 | Training | 5.2218 | 5.4112 | 0.1894 |
| 22 | Training | 5.8239 | 5.9432 | 0.1193 |
| 23 | Prediction | 6.3665 | 6.7304 | 0.3639 |
| 24 | Prediction | 6.5376 | 6.3979 | -0.1397 |
| 25 | Prediction | 5.6198 | 4.7751 | -0.8447 |
| 26 | Training | 6.3279 | 6.6658 | 0.3379 |
| 27 | Training | 5.3665 | 5.3443 | -0.0222 |
| 28 | Training | 6.5528 | 6.5846 | 0.0318 |
| 29 | Training | 5.6198 | 5.3549 | -0.2649 |
| 30 | Prediction | 5.4318 | 5.1436 | -0.2882 |
| 31 | Training | 5.4318 | 5.4364 | 0.0046 |
| 32 | Training | 5.3372 | 5.5874 | 0.2502 |
| 33 | Prediction | 5.7696 | 5.5629 | -0.2067 |
| 34 | Training | 5.2676 | 5.2647 | -0.0029 |
| 35 | Prediction | 6.2076 | 6.3391 | 0.1315 |
| 36 | Training | 6.4815 | 6.686 | 0.2045 |
| 37 | Prediction | 6.3468 | 6.2226 | -0.1242 |
| 38 | Training | 6.8861 | 6.6982 | -0.1879 |
| 39 | Training | 6.7959 | 6.7215 | -0.0744 |
| 40 | Prediction | 7.0177 | 6.6783 | -0.3394 |
| 41 | Training | 5.5376 | 5.4602 | -0.0774 |
| 42 | Training | 5.6576 | 5.4534 | -0.2042 |
| 43 | Training | 5.4318 | 5.3729 | -0.0589 |
| 44 | Training | 5.7959 | 5.8108 | 0.0149 |
| 45 | Training | 7.6198 | 6.6601 | -0.9597 |
| 46 | Training | 6.8539 | 6.7522 | -0.1017 |
| 47 | Prediction | 6.7696 | 6.6982 | -0.0714 |
| 48 | Training | 5.3565 | 5.3333 | -0.0232 |
| 49 | Prediction | 5.3565 | 5.2194 | -0.1371 |
| 50 | Training | 6.6383 | 6.6982 | 0.0599 |
| 51 | Prediction | 6.3098 | 6.5253 | 0.2155 |
| 52 | Training | 6.7959 | 6.4464 | -0.3495 |
| 53 | Training | 6.2676 | 6.246 | -0.0216 |
| 54 | Training | 6.2757 | 6.6831 | 0.4074 |
| 55 | Training | 5.585 | 5.1083 | -0.4767 |
| 56 | Training | 6.3279 | 6.6607 | 0.3328 |
| 57 | Training | 6.3098 | 6.2766 | -0.0332 |
| 58 | Training | 5.4437 | 6.1034 | 0.6597 |
| 59 | Training | 5.4089 | 5.4864 | 0.0775 |
| 60 | Prediction | 5.585 | 5.521 | -0.064 |
| 61 | Training | 5.0706 | 5.4409 | 0.3703 |
| 62 | Training | 5.1871 | 5.4409 | 0.2538 |
| 63 | Training | 5.4685 | 5.5071 | 0.0386 |

|   Pharm1 |   Pharm2 |   Pharm3 |
| --- | --- | --- |
|   Pharm4 |   Pharm5 |   Pharm6 |
|   Pharm7 | |   Pharm8 |
|   Pharm9 |   Pharm10 |   Pharm11 |
|   Pharm12 |   Pharm13 |   Pharm14 |
|   Pharm15 |   Pharm16 |   Pharm17 |
|   Pharm18 |   Pharn19 |   Pharm20 |
|   Pharm21 |   Pharm22 |   Pharm23 |
|   Pharm24 |   Pharm25 |   Pharm26 |
|   Pharm27 |   Pharm28 |   Pharm29 |
|   Pharm30 |   Pharm31 |   Pharm32 |
|   Pharm33 |   Pharm34 | |
|   Pharm35 |   Pharm36 |   Pharm37 |
|   Pharm38 |   Pharm39 |   Pharm40 |

Fig. S1. The generated compounds were through pharmacophore modeling with the Pharmit web server.
